# Tumor-associated macrophages promote Ezrin phosphorylation-mediated epithelial-mesenchymal transition in lung adenocarcinoma through FUT4/LeY up-regulation

**DOI:** 10.18632/oncotarget.16001

**Published:** 2017-03-08

**Authors:** Aman Wang, Chang Lu, Zhen Ning, Wei Gao, Yunpeng Xie, Ningning Zhang, Jinxiao Liang, Faisal S. Abbasi, Qiu Yan, Jiwei Liu

**Affiliations:** ^1^ Department of Oncology, The First Affiliated Hospital of Dalian Medical University, 116011, Dalian, China; ^2^ Department of Hepatobiliary Surgery, The First Affiliated Hospital of Dalian Medical University, 116011, Dalian, China; ^3^ City College, Zhejiang University, 310000, Hangzhou, China; ^4^ Department of Cardiology, The First Affiliated Hospital of Dalian Medical University, 116011, Dalian, China; ^5^ Department of Thoracic Surgery, Zhejiang Cancer Hospital, 310000, Hangzhou, China; ^6^ Department of Biochemistry and Molecular Biology, Dalian Medical University, 116011, Dalian, China

**Keywords:** tumor-associated macrophages, Ezrin, epithelial-mesenchymal transition, FUT4, LeY

## Abstract

Tumor-associated macrophages (TAMs) are key components of tumor microenvironment (TME) during tumorigenesis and progression. However, the role of TAMs in lung adenocarcinoma is still unclear. In this study, we aimed to clarify the mechanism underlying the crosstalk between TAMs and epithelial-mesenchymal transition (EMT) of lung adenocarcinoma. Fucosyltransferase IV (FUT4) and its synthetic cancer sugar antigen Lewis Y (LeY) was aberrantly elevated in various solid tumors, it plays critical role in the invasion and metastasis. Here, we found that in lung adenocarcinoma samples, the density of TAMs correlates with E-cadherin level and LeY level. *In vitro* assays, M2 macrophages promoted FUT4/LeY expression through the transforming growth factor-β1(TGF-β1)/Smad2/3 signaling pathway. FUT4/LeY was indispensable in M2 macrophages-mediated cytoskeletal remodeling and EMT. Furthermore, fucosylation of Ezrin mediated by FUT4/LeY can promote the phosphorylation of Ezrin, which was the critical mechanism of M2 macrophages-induced EMT. *In vivo* assays confirmed that M2 macrophages promoted EMT through the up-regulation of LeY and phosphorylated Ezrin. Together, our results revealed that TAMs promote Ezrin phosphorylation-mediated EMT in lung adenocarcinoma through FUT4/LeY- mediated fucosylation. Targeting this newly identified signaling may offer new possibilities for immunotherapy in lung adenocarcinoma.

## INTRODUCTION

Non–small cell lung cancer (NSCLC), which accounts for 80% to 85% of all lung cancers, is the most fatal cancer worldwide, with a 5-year survival as low as 13% [1]. Recent evidence suggests the tumor microenvironment (TME) is critical for the initiation, progression and metastasis of tumor [2]. Macrophages infiltrated in the tumor stroma are define as tumor-associated macrophages (TAMs), and they are the most abundant inflammatory cells in the TME [3]. TAMs are derived from circulating monocytes and recruited at the tumor site by various chemo attractants, providing a key link between inflammation and cancer [4]. TAMs exhibit the macrophages M2 polarization phenotype and enhance tumor angiogenesis, growth, metastasis, and immunosuppression by secreting a series of cytokines, chemokines, and proteases [5]. Several studies have revealed that an increased density of TAMs was correlated with poor prognosis in various cancers including NSCLC [6]. This association is linked to the typical presence of the M2-polarized TAMs during the process of tumor invasion, however, the exact mechanisms underlying the process is still not clear.

Epithelial-mesenchymal transition (EMT) is a process, in which epithelial tumor cells lose epithelial feather and gain mesenchymal phenotypes [7]. It is considered as the critical step by which tumor cells gain the higher capabilities of invasion and metastasis. During this process, epithelial cells lose polarity, the cell adhesion decreases, cytoskeletal remodeling occurs in these cells and provides mobility and plasticity, the expression of the epithelial marker E-cadherin decreases, and the expression of the stromal marker N-cadherin and transcription factor Snail increases [8]. Previous studies have demonstrated that EMT was correlated with carcinogenesis, metastasis, and poor prognosis in various types of cancers including NSCLC [9]. Recent study suggests that, TAMs play a protumor role and promote EMT through activation of growth arrest-specific gene 6 (Gas6)/Axl and nuclear factor (NF)-κB signaling in oral squamous cell carcinoma [10]. M2-polarized TAMs promote EMT in pancreatic cancer cells through Toll-like receptor 4 (TLR4)/interleukin-10 (IL-10) signaling [11]. TAMs can induce EMT in intratumoral cancer cells through transforming growth factor-β (TGF-β) signaling and activation of the β-catenin pathway [12]. These confirm a strong crosstalk between macrophages and tumor progression, mainly through stimulation of EMT.

Glycosylation is a common post-translational regulation event during the process of eukaryotic gene expression. Abnormal glycosylation is a characteristic feature of tumors and is associated with the abnormal expression of glycosyltransferase enzymes [13]. The Lewis oligosaccharide belongs to the A, B, and H blood type antigen family. One family member, LeY is a type of difucosylated oligosaccharide and its molecular structure is [Fuca1-2Galb1-4(Fuca1-3) GlcNAcb1-R]. FUT4 catalyzes the transfer of the Fuc of GDP-Fuc to the N-acetylglucosamine of the sugar chain, and FUT4 is a key enzyme in the synthesis of the tumor-associated carbohydrate antigen LeY. Recent studies and our previous work revealed that FUT4/LeY was highly expressed in a variety of solid tumors including breast cancer, ovarian cancer, pancreatic cancer, colon cancer, melanoma and NSCLC and was associated with invasion, metastasis, and poor prognosis [14–18]. Our recent study also showed that FUT4/LeY could activate epidermal growth factor receptor (EGFR) and promote the EMT, invasion, and metastasis of NSCLC through mitogen-activated protein kinase (MAPK)/NF-κB pathway [19]. However, few studies have evaluated the N-fucosylation mediated by FUT4/LeY in lung adenocarcinoma; in addition, there is no report on the relationship between fucosylation and immune cells in the TME.

In this study, we aim to analyze the role of FUT4/LeY-mediated fucosylation in TAMs-induced EMT in lung adenocarcinoma. We found that in lung adenocarcinoma samples, the density of TAMs correlates with E-cadherin level and LeY level. *In vitro* assays, M2 macrophages promoted FUT4/LeY expression through the TGF-β1/Smad2/3 signaling pathway. FUT4/LeY was indispensable in M2 macrophages-mediated cytoskeletal remodeling and EMT. Furthermore, fucosylation of Ezrin mediated by FUT4/LeY can promote the phosphorylation of Ezrin, which was the critical mechanism of M2 macrophages-induced EMT. *In vivo* assays confirmed that M2 macrophages promoted EMT through the up-regulation of LeY and phosphorylated Ezrin. We demonstrated that TAMs promote Ezrin phosphorylation-mediated EMT in lung adenocarcinoma through FUT4/LeY- mediated fucosylation.

## RESULTS

### The density of TAMs correlates with E-cadherin level and LeY level in human lung adenocarcinoma tissues

**Figure 1:**
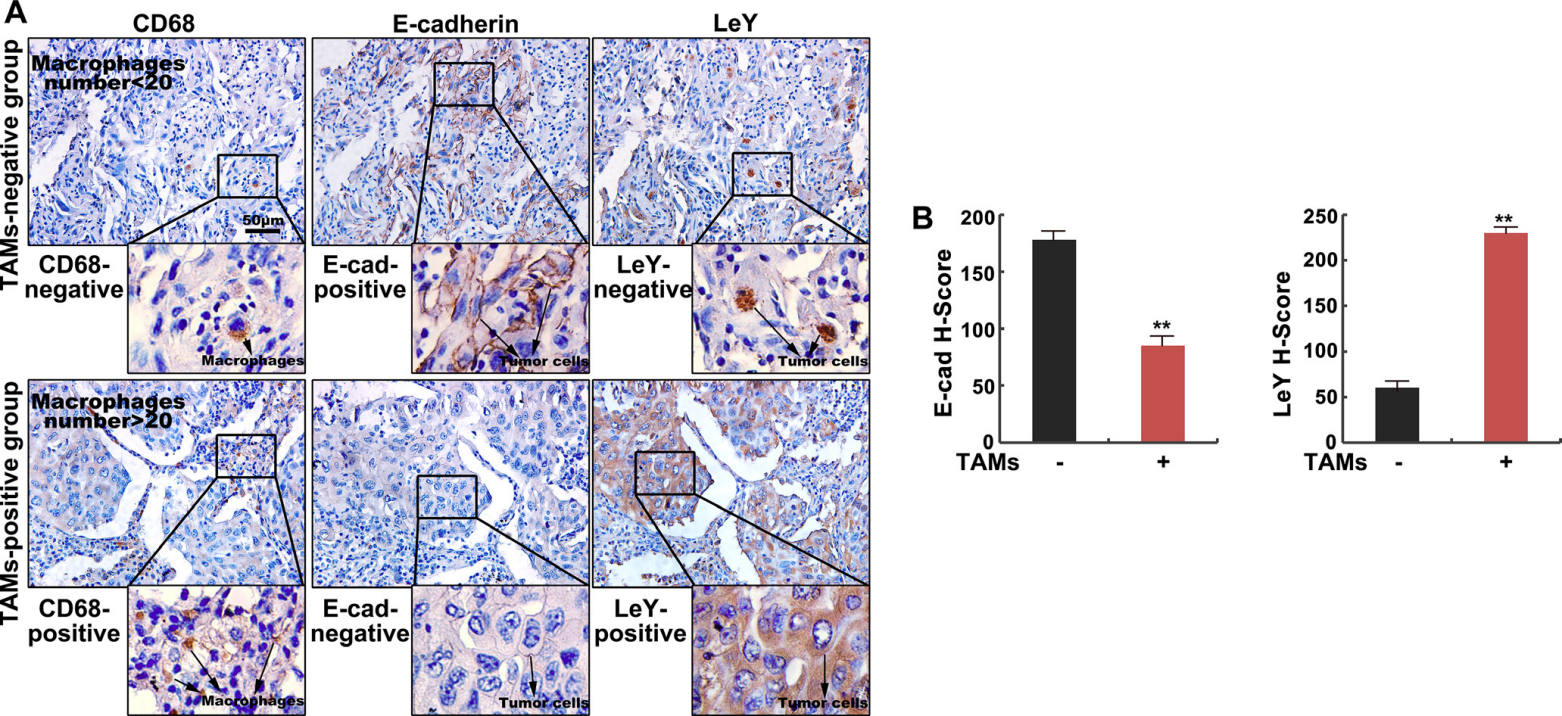
The density of TAMs correlates with E-cadherin level and LeY level in human lung adenocarcinoma tissues (**A**) Immunohistochemical staining of E-cadherin, LeY and CD68 (macrophages) in human lung adenocarcinoma specimens. Shown are two representative specimens (bar = 50 μm). (**B**) The staining intensity of E-cadherin and LeY in TAM (−) and TAM (−) groups by H-score.

To assess the relation of TAMs-mediated EMT and LeY, we performed immunohistochemistry in 60 human lung adenocarcinoma specimens. The expression of CD68 (a marker of human macrophages) and LeY was shown in cell membrane and cytoplasm and developed into a brown color. The expression of E-cadherin was mainly concentrated in the cell membrane with minute staining in the cytoplasm and developed into a brown color (Figure 1A). As shown in Figure 1A and 1B, the staining intensity of E-cadherin in the TAMs negative group (CD68 negative) was higher than that in the TAMs positive group (CD68 positive) (*P* < 0.01), while LeY expression was lower than that in the TAMs positive group (*P* < 0.01). The relationships between the expression of CD68, E-cadherin and LeY were calculated and have been outlined in Table 1. The result showed that the positive expression of CD68 correlated with a loss of E-cadherin expression (r = −0.505, *P* < 0.01) and positive expression of LeY (r =0.55, *P* < 0.01). As shown in Table 2, there is a negative correlation between the expression of E-cadherin and LeY (r = −0.798, *P* < 0.01). Taken together, these results demonstrate that the density of TAMs correlates with E-cadherin level and LeY level in human lung adenocarcinoma.

**Table 1:** The association between the expression of CD68, E-cadherin and LeY in human lung adenocarcinoma tissues

	CD68		*r*	*P*
	positive	negative		
**E-cadherin**				
** positive**	4	14	−0.505	< 0.001
** negative**	32	10		
**LeY**				
** positive**	34	11	0.55	< 0.001
** negative**	2	13		

**Table 2:** The association between the expression of E-cadherin and LeY in human lung adenocarcinoma tissues

	LeY		*r*	*P*
	positive	negative		
**E-cadherin**				
** positive**	4	14	−0.798	< 0.001
** negative**	41	1		

### M2 macrophages promoted FUT4/LeY expression through the TGF-β1/Smad2/3 signaling pathway in *in vitro* assays

**Figure 2:**
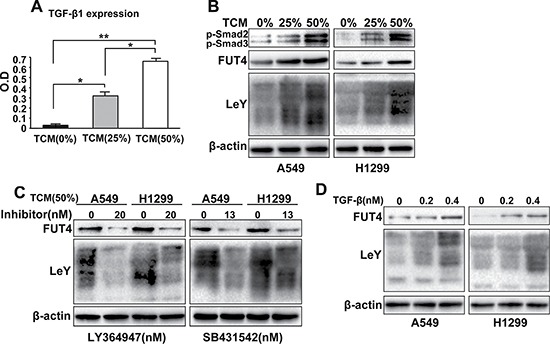
M2 macrophages promoted FUT4/LeY expression through the TGF-β1/Smad2/3 signaling pathway in *in vitro* assays **(A)** TGF-β1 level in TCM was measured by ELISA. **(B)** A549 and H1299 cells were treated with TCM in different concentration(0%,25% or 50%) for 48h. The expression of p-Smad2/3, FUT4 and LeY were analyzed by western blot analysis. **(C)** A549 and H1299 cells were treated with TCM (50%) and the TGF-β1/Smad2/3 signaling pathway inhibitors, LY364947 or SB431542, respectively. The expression of FUT4 and LeY were analyzed by western blotting. **(D)** A549 and H1299 cells were treated with different concentrations of TGF-β1. The expression of FUT4 and LeY were analyzed by western blot analysis. Every experiment was conducted at least three times, and the average is shown (mean ± SD). *P < 0.05, **P < 0.01, significant.

TAMs exhibit an M2-like phenotype, which are characterized by high secretion of a variety of cytokines including TGF-β1. TGF-β1 has been previously accepted to play a major role in tumor progression via EMT. To identify TAMs-derived factors that enhance LeY expression in lung adenocarcinoma, we prepared the M2 macrophages conditioned medium (TAMs conditioned medium, TCM). ELISA assay showed that the TGF-β1 expression was elevated in TCM (Figure 2A). As shown in Figure 2B, TCM significantly promoted FUT4/LeY expression and Smad2/3 phosphorylation in lung adenocarcinoma cell lines (A549 and H1299) in a dose-dependent manner. In addition, we applied two TGF-β1/Smad2/3 signaling pathway inhibitors, LY364947 and SB431542, to separately pretreated A549 and H1299 cells. When A549 and H1299 cells were incubated with TCM and treated with LY364947 and SB431542, FUT4/LeY expression was partially suppressed (Figure 2C). In further experiments, we applied different concentrations of TGF-β1 to A549 and H1299 cells and showed that TGF-β1 had significant dose-dependent effects on the FUT4/LeY expression (Figure 2D). On the basis of these findings, we concluded that M2 macrophages could promote FUT4/LeY expression in lung adenocarcinoma cells through the TGF-β1/Smad2/3 signaling pathway.

### FUT4/LeY was indispensable in M2 macrophages-mediated cytoskeletal remodeling and EMT

**Figure 3:**
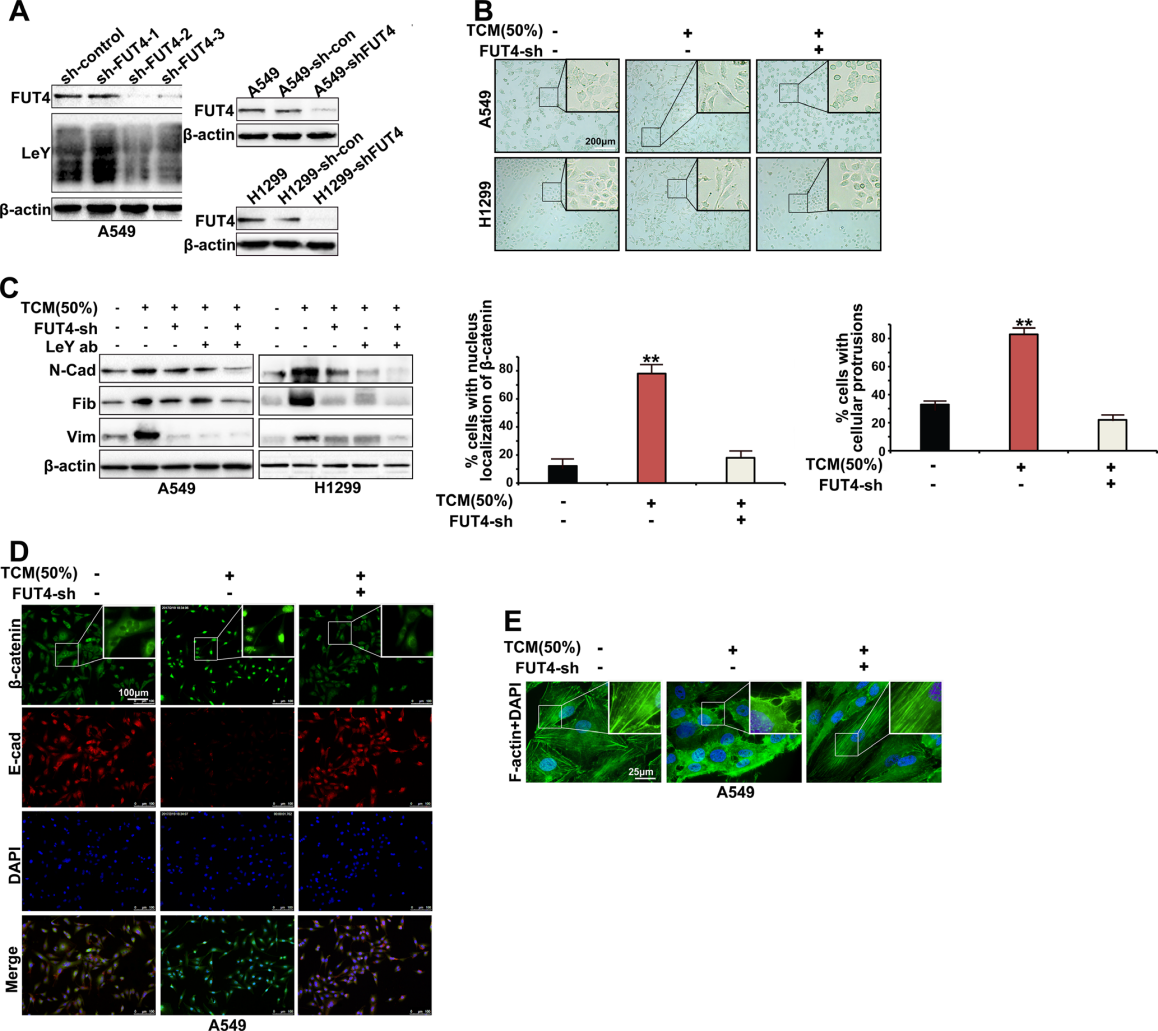
FUT4/LeY was indispensable in M2 macrophages-mediated cytoskeletal remodeling and EMT (**A**) A549 and H1299 cells were transfected with FUT4 shRNA for 72 h. The expression of FUT4 and LeY were analyzed by western blotting. After G418 selection, the cell clones sh-FUT4-2 and sh-con, which were stably transfected with the corresponding plasmids, were selected for future studies. (**B**) A549/H1299 cells and A549-shFUT4/H1299-shFUT4 cells were treated with indicated dose of TCM for 48 h. The cancer cells were analyzed by microscopy (original magnification, bar = 200 μm). (**C**) A549/H1299 cells and A549-shFUT4/H1299-shFUT4 cells were preincubated with immunoglobulin G (IgG) or LeY blocking antibody for 1 h. Then cells were stimulated with indicated dose of TCM for 48 h. The expression of N-cadherin, Vimentin and Fibronectin were analyzed by western blot analysis. (**D**) A549 cells and A549-shFUT4 cells were treated with indicated dose of TCM for 48 h. E-cadherin and β-catenin expression was detected by immunofluorescence staining (bar = 100 μm). The cells with nucleus localization of β-catenin were counted. (**E**) A549 cells and A549-shFUT4 cells were treated with indicated dose of TCM for 48 h. F-actin was detected by immunofluorescence staining (bar = 25 μm). The cells with extended cellular protrusions were counted. Every experiment was conducted at least three times, and the average is shown (mean ± SD). *P < 0.05, **P < 0.01, significant.

To further clarify the role of FUT4/LeY up-regulation in TAMs-mediated EMT, we stably eliminated the FUT4 expression in A549 and H1299 cells and used G418 to screen the cell clones (Figure 3A). As shown in Figure 3B, after TCM induction, the morphology of A549 and H1299 cells changed from a pebble-like shape to an elongated shape, which exhibited the mesenchymal cell phenotype. Western blot analysis also showed the increased expression of mesenchymal markers N-cadherin, Vimentin and Fibronectin (Figure 3C). Significantly, upon FUT4 down-regulation by RNAi, the morphology of A549 and H1299 cells no longer change significantly after TCM treatment, and the expression of mesenchymal markers N-cadherin, Vimentin and Fibronectin significantly decreased (Figure 3B and 3C). In addition, after down-regulation of LeY through either FUT4 interference or the use of exogenous LeY antibodies to directly block the LeY antigens on the surface of A549 cells, TCM could not effectively promote the EMT process in A549 and H1299 cells (Figure 3C). Immunofluorescent staining showed that under TCM induction, the epithelial marker E-cadherin of A549 cells was significantly down-regulated, and β-catenin was accumulated and translocated from the cell membrane and cytoplasm into the nucleus. After FUT4 down-regulation by RNAi, E-cadherin expression levels gradually increased and β-catenin relocalized to the cell membrane and cytoplasm (Figure 3D).

Cytoskeletal remodeling is an important stage in the process of EMT. Then we performed immunofluorescence assays to examine the cytoskeletal structure in A549 cells in different conditions. As shown in Figure 3E, in the control A549 cells, the F-actin was distributed as a net representing of normal cytoskeletal structure. After TCM induction, F-actin cytoskeleton was altered with increased polymerization and extended cellular protrusions. After FUT4 down-regulation by RNAi, TCM no longer elicited the above changes of F-actin (Figure 3E). Collectively, the data revealed that FUT4/LeY was indispensable in M2 macrophages-mediated cytoskeletal remodeling and vEMT.

### FUT4/LeY-mediated fucosylation of Ezrin was closely associated with the phosphorylation of Ezrin

**Figure 4:**
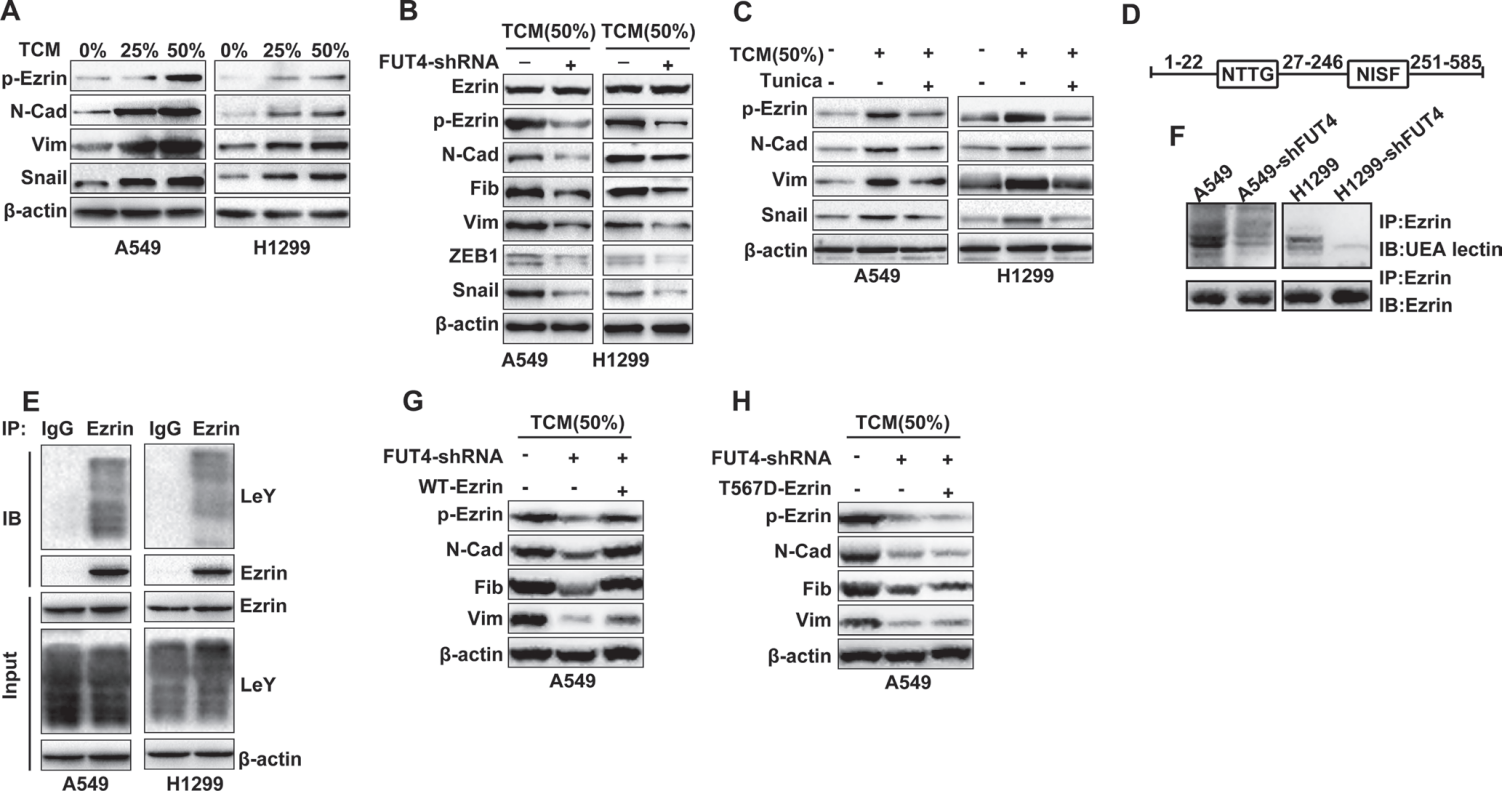
FUT4/LeY-mediated fucosylation of Ezrin was closely associated with the phosphorylation of Ezrin (**A**) A549 and H1299 cells were treated with TCM in different concentration (0%–25% or 50%) for 48 h. The expression of p-Ezrin (T567), N-cadherin, Vimentin and Snail were analyzed by western blot analysis. (**B**) A549/H1299 cells and A549-shFUT4/H1299-shFUT4 cells were treated with indicated dose of TCM for 48 h. The expression of Ezrin, p-Ezrin (T567), N-Cadherin, Fibronectin, Vimentin, ZEB1 and Snail were analyzed by western blot analysis. (**C**) A549 and H1299 cells preincubated with tunicamycin for 4 h. Then cells were stimulated with indicated dose of TCM for 24 h. The expression of p-Ezrin (T567), N-cadherin, Vimentin and snail were analyzed by western blot analysis. (**D**) Schematic diagram of the potential N-glycosylation sites on the Ezrin protein. (**E**) Cell lysates were immunoprecipitated with either IgG or Ezrin antibody and detected by western blot analysis as indicated. (**F**) Cell lysates were immunoprecipitated using Ezrin antibody, and the samples were separated using SDS-PAGE followed by blotting biotinylated UEA lectin or Ezrin antibody. (**G**) A549 cells and A549-shFUT4 cells were transfected with WT Ezrin plasmids or vector. Then cells were stimulated with indicated dose of TCM for 48 h. The expression of p-Ezrin (T567), N-cadherin, Vimentin and Fibronectin were analyzed by western blot analysis. (**H**) A549 cells and A549-shFUT4 cells were transfected with T567D-mutant Ezrin plasmids or vector. Then cells were stimulated with indicated dose of TCM for 48 h. The expression of p-Ezrin (T567), N-cadherin, Vimentin and Fibronectin were analyzed by western blot analysis. Every experiment was conducted at least three times, and the average is shown (mean ± SD). *P < 0.05, **P < 0.01, significant.

As an important regulatory protein, Ezrin links cell membrane molecules (CD44 and ICAM-2) to actin. After activation, phosphorylated Ezrin plays critical roles in cytoskeletal remodeling and the TGF-β-mediated EMT. As shown in Figure 4A, during the EMT induced by TCM, the Ezrin phosphorylation (T567) increased significantly in a dose-dependent manner in A549 and H1299 cells, which is similar to the FUT4/LeY expression in Figure 2B. However, after down-regulation of LeY through FUT4 interference, the Ezrin phosphorylation (T567) decreased significantly, which was accompanied by the decreased expression of mesenchymal markers (Figure 4B).

To verify the TAMs-induced fucosylation of A549 cells, we used tunicamycin as pretreatment to inhibit N-glycosylation modification in A549 and H1299 cells. Western blots analysis showed that the Ezrin phosphorylation (T567) induced by TCM was inhibited after tunicamycin pretreatment, which was also accompanied by the decreased expression of mesenchymal markers (Figure 4C).

To further elucidate the underlying mechanism, we performed bioinformatics analysis (http://www.cbs.dtu.dk/services/NetNGlyc/) to identify the potential N-glycosylation sites on the Ezrin protein. The data showed that Ezrin protein was composed of 585 amino acids, and the sites of 23-26 and 247-250 were the potential N-glycosylation sites of Ezrin (Figure 4D). In addition, co-immunoprecipitation result showed that Ezrin protein indeed had a certain amount of LeY glycoproteins (Figure 4E). We next compared fucosylated Ezrin between A549/H1299 and A549-shFUT4/H1299-shFUT4 (A549/H1299 cells with the stable depletion of FUT4) cells using lectin blotting with UEA lectin, which recognizes fucosylation on N-glycans. The fucosylation of Ezrin was decreased in A549-shFUT4/H1299-shFUT4 cells, compared with that in A549/H1299 cells (Figure 4F).

To confirm the specific effect of FUT4/LeY on the Ezrin phosphorylation in EMT process, we constructed the WT and T567D-mutant Ezrin vector and transfected them into A549-shFUT4 cells. Western blots analysis showed that the down-regulation of Ezrin phosphorylation and mesenchymal markers after knockdown of FUT4 can be counteracted by the overexpression of WT Ezrin but not T567D-mutant Ezrin (Figure 4G and 4H). These results suggested that FUT4/LeY-mediated fucosylation of Ezrin was closely associated with the phosphorylation of Ezrin.

### *In vivo* assays confirmed that M2 macrophages promoted EMT through the up-regulation of LeY and phosphorylated Ezrin

**Figure 5:**
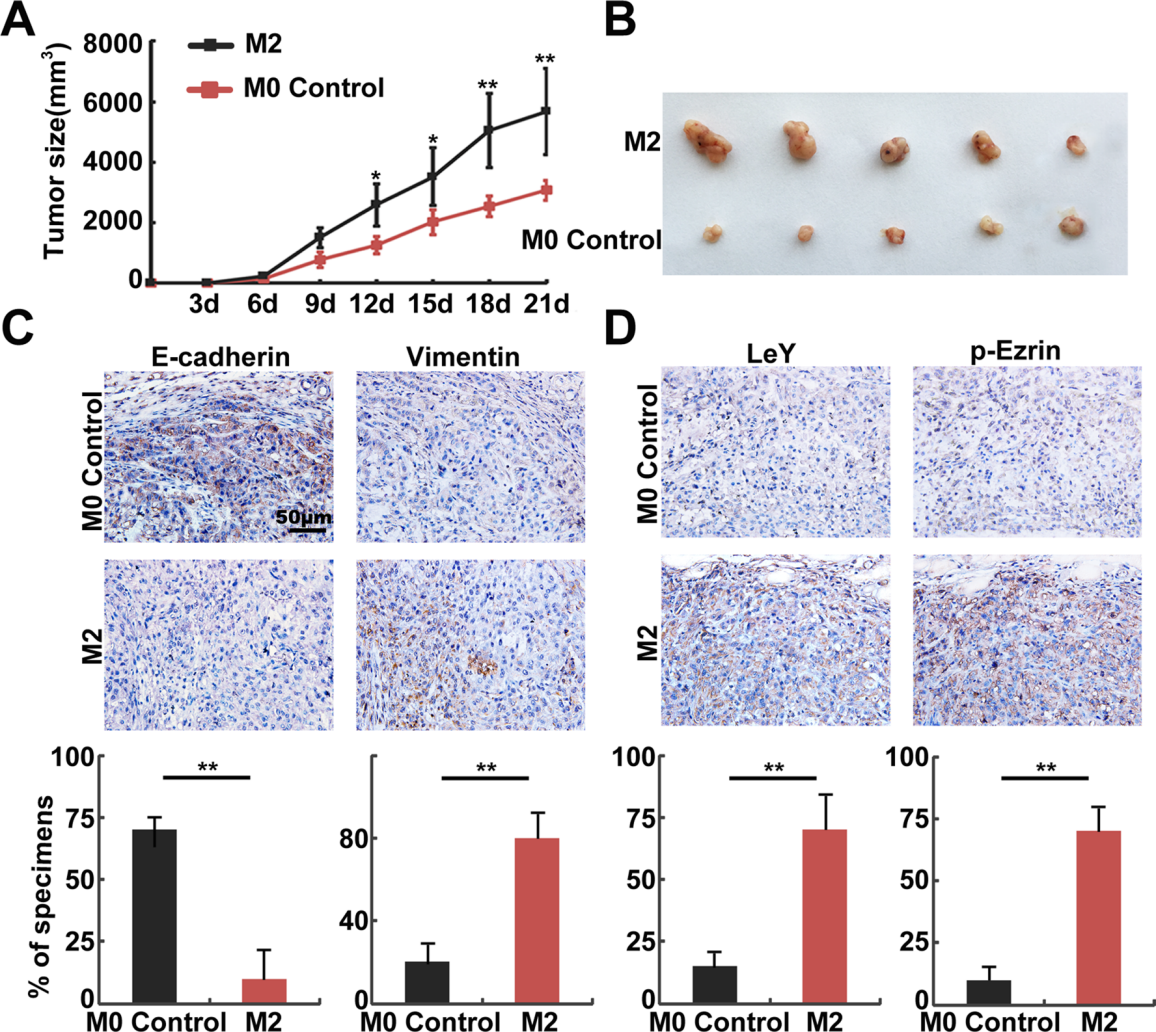
*In vivo* assays confirmed that M2 macrophages promoted EMT through the up-regulation of LeY and phosphorylated Ezrin (**A**) Growth curves of mammary tumors after M2 or M0 control macrophages were pre-mixed with A549 cells at a 1:4 ratio and were subcutaneously implanted into nude mice. (**B**) The volume of tumors in the M2 group were higher than those in the M0 control group. (**C** and **D**) Representative examples of E-cadherin, Vimentin, LeY, and p-Ezrin immunohistochemistry from the xenografts (bar = 50 μm).

To further investigate the relation of LeY, phosphorylated Ezrin and TAMs-mediated EMT, M2 or M0 macrophages were pre-mixed with A549 cells at a 1:4 ratio and were subcutaneously implanted into nude mice [20]. The M2 group (M2 macrophages and A549 co-injection) already had tumor formation on day 6; in contrast, the M0 control group (M0 macrophages and A549 co-injection) had tumor formation on day 11. The volume (5933 ± 568 mm^3^ VS 3240 ± 246 mm^3^, *P* < 0.01) of the tumors in the M2 group were also higher than those in the M0 control group (Figure 5A and 5B). Mice were sacrificed after 21 days, and tumor tissues were removed and embedded in paraffin. The results of subsequent immunohistochemistry experiments showed that in the serial sections, the E-cadherin expression at the cell membrane in the M2 group was significantly lower than that in the M0 control group, which was accompanied by up-regulation of Vimentin (Figure 5C). We also performed immunohistochemistry using LeY and p-Ezrin antibodies, and the results showed significantly higher expression of LeY and p-Ezrin in the M2 group than in the M0 control group (Figure 5D). These results confirmed that M2 macrophages promoted EMT through the up-regulation of LeY and phosphorylated Ezrin.

## DISCUSSION

The critical role of TME in modulating cancer initiation, invasion and metastasis has been widely accepted. As a main component of tumor-infiltrating leukocyte in TME, TAMs play a decisive role in tumor progression through the release of cytokines, chemokines, growth factors, and matrix metalloproteases [21]. To date, few studies have attempted to demonstrate the complex interactions between TAMs and NSCLC, a better understanding of underlying mechanism of TAMs regulate the progression of NSCLC was needed.

Several studies showed that TAMs are polarized to M2 phenotype in the TME and promote cancer cell growth, invasion, and metastasis [3]. Here we used the M2 macrophages, derived from THP-1, as a substitute to explore the impact of TAMs on the lung adenocarcinoma cells. CD68, a highly glycosylated lysosomal membrane protein, which is strongly expressed in cytoplasm of macrophages, was widely used as a marker to identify TAMs in tissues in several studies [22, 23].

EMT is a key process of the cancer cells dissemination and metastasis [24]. By suppressing the expression of epithelial markers such as E-cadherin, increasing the expression of mesenchymal markers including N-cadherin, Vimentin, Slug, Snail, Fibronectin, ZEB1, and enhancing the nuclear translocation of β-catenin, the cells acquire the ability to migrate and invade, which could lead to tumor progression and metastasis [25]. Here we determined the expression of CD68 and E-Cadherin in 60 lung adenocarcinoma tissues using immunohistochemistry. In agreement with previous results, our results showed that the positive expression of CD68 correlated with a loss of E-cadherin expression (Figure 1A, 1B and Table 1). The subsequent *in vitro* experiments showed that A549 and H1299 cells treated by TCM underwent EMT, accompanied with alterations in morphology toward an elongated shape, an increase in the expression of mesenchymal markers and nuclear translocation of β-catenin (Figure 3B, 3C and 3D). These results indicate that TAMs can promote EMT in lung adenocarcinoma.

What is the mechanism of M2-polarized TAMs-mediated EMT in lung adenocarcinoma cells? Abnormal glycosylation plays key roles in all stages of the malignant transformation, invasion, and metastasis and is a characteristic feature of tumors [26]. Studies have revealed that abnormal glycosylation closely correlated with EMT [27–29]. Fucosylation is an important post-translational modification and functional regulation of glycoproteins and is critical for the abnormal glycosylation that is found in tumors. In our previous study at our laboratory, it was shown that FUT4 and its regulated LeY sugar chains were significantly up-regulated in NSCLC and breast cancer and promoted EMT induction through EGFR and PI3K/Akt-GSK3β activation[19, 30]. However, there is no report on whether abnormal glycosylation is an important link in the EMT mediated by TAMs. As shown in this study, the immunohistochemistry results in lung adenocarcinoma tissues revealed that the positive expression of CD68 correlated with a loss of E-cadherin expression and positive expression of LeY (Figure 1A and B, Table 1). There is a negative correlation between the expression of E-cadherin and LeY (Table 2). *In vitro*, experiments showed that M2 macrophages could promote EMT in lung adenocarcinoma cells. However, after down-regulation of LeY expression by FUT4 silencing or direct blocking of LeY antigens on the cell surface using LeY antibodies, M2 macrophages could not effectively induce the EMT in A549 and H1299 cells (Figure 3B, 3C and 3D). These results demonstrate that the up-regulation of FUT4/LeY was critical in TAMs-mediated EMT.

Cytokines are important mediators of the tumor-promoting functions of TAMs [31]. As one of the most important members of TGF-β, TGF-β1 plays critical roles in cancer progression and metastasis, which induces EMT in cancer cells [32–34]. Recent findings provided convincing evidence that there was a tight crosstalk between TAMs and TGF-β1 signaling pathways, and TAMs may play a central role in EMT through interaction with TGF-β1 signaling [35, 36]. Our study showed that TGF-β1 expression was elevated in TCM (Figure 2A). Previous study reported that TGF-β1 expression correlates with LeY expression in hepatocellular carcinoma by immunohistochemistry [37]. Our results showed that M2 macrophages could significantly promote FUT4/LeY expression in lung adenocarcinoma cell lines (Figure 2B). However, M2 macrophages could not complete this process after the TGF-β1/Smad2/3 signaling pathway was blocked (Figure 2C). These results demonstrate that TAMs promoted FUT4/LeY expression through the TGF-β1/Smad2/3 signaling pathway.

Cytoskeletal remodeling is an important step in EMT [25, 38]. The cytoskeleton is a sub-membranous fibrillar network of microtubules, actin microfilaments, intermediate filaments, and myosin filaments. Changes in the structure and function of the cytoskeleton are controlled by the remodeling of the actin-based microfilament cytoskeleton. Cells use actin filaments to form protrusions (pseudopodia and filopodia) that drive cell motility [39]. Abundant expression of filopodia is often observed on the membrane of invasive cancer cells [40]. As shown in our study, after TCM induction, F-actin cytoskeleton was altered with increased polymerization and extended cellular protrusions. After down-regulation of FUT4, TCM no longer elicited the above changes of F-actin (Figure 3E). These results revealed that FUT4/LeY was indispensable in M2 macrophages-mediated cytoskeletal remodeling.

Ezrin is an important regulatory protein, and it links cell membrane molecules (CD44 and ICAM-2) to actin[41]. Ezrin overexpression is associated with the metastasis of a variety of solid tumors [42]. Previous study reported that Ezrin played critical roles in the TGF-β1-mediated EMT process in A549 cells [43]. After activation, phosphorylated Ezrin can participate in a variety of actin-dependent biological functions. Phosphorylation and dephosphorylation of Ezrin at T567 has been identified as a critical step in the conformational activation and deactivation of Ezrin [44]. Our previous studies confirmed that phosphorylated Ezrin could mediate EMT in pancreatic cancer cells through the FAK pathway and promote invasion and metastasis [45]. In this study, during the EMT induced by TCM, Ezrin phosphorylation (T567) increased significantly (Figure 4A), which is similar to the expression of FUT4/LeY (Figure 2B). After down-regulation of LeY through FUT4 interference, Ezrin phosphorylation (T567) decreased significantly, which was accompanied by the decreased expression of mesenchymal markers (Figure 4B). To further verify the relation between TAMs-induced fucosylation and Ezrin phosphorylation, we performed bioinformatics analysis and found that there were two potential N-glycosylation sites on the Ezrin protein. Co-immunoprecipitation result showed that Ezrin protein indeed had a certain amount of LeY glycoproteins. After down-regulation of LeY through FUT4 interference, the fucosylation of Ezrin was decreased significantly (Figure 4D, 4E and 4F). These results preliminarily confirmed that, in the process of post-translational modifications, the fucosylation of Ezrin occurred. It is reported in previous study that dynamic regulation of Ezrin phosphorylation at amino acid T567 that controls structural transitions of this protein plays a pivotal role in tumor progression and metastasis [44]. To confirm the specific effect of FUT4/LeY on the Ezrin phosphorylation in EMT process, we down-regulated FUT4 by interference and found that Ezrin phosphorylation and EMT can be counteracted by the overexpression of WT Ezrin but not T567D-mutant Ezrin. These results suggested that FUT4/LeY mediated fucosylation of Ezrin was closely associated with the phosphorylation of Ezrin (T567). Our *in vivo* experiments confirmed that M2 macrophages could significantly up-regulate the expression of Vimentin, LeY and p-Ezrin and down-regulate the expression of E-cadherin, thus promote the growth and EMT of lung adenocarcinoma xenograft. These results further confirmed our previous conclusion.

**Figure 6:**
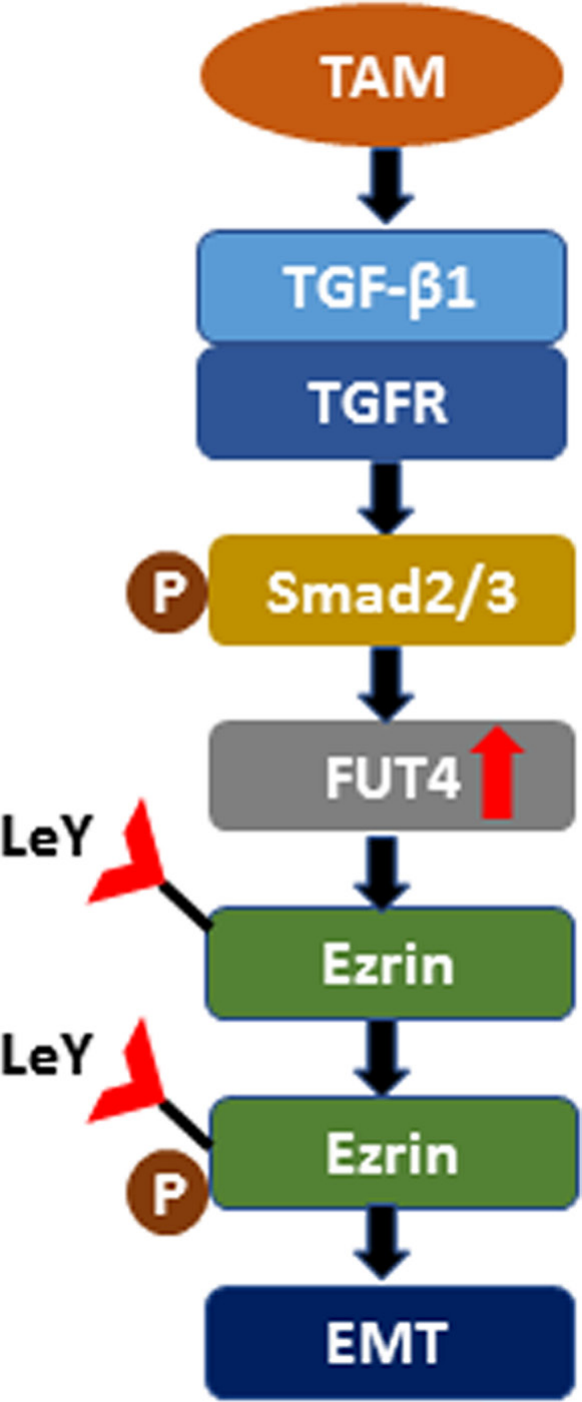
Schematic diagram shows the mechanism that TAMs promote Ezrin phosphorylation mediated EMT in lung adenocarcinoma through FUT4/LeY -mediated fucosylation

In summary, sufficient evidence from *in vivo* and *in vitro* experiments indicated that FUT4/LeY was critical to the TAMs-mediated EMT; this process might be associated with the up-regulation of Ezrin phosphorylation by FUT4/LeY-mediated fucosylation (Figure 6). Thus, the blockade of the polarization of macrophages in tumors through targeting of FUT4/LeY is expected to provide new possibilities for immunotherapy in lung adenocarcinoma.

## MATERIALS AND METHODS

### Patients and tissue samples

Paraffin-embedded specimens were obtained from 60 patients with lung adenocarcinoma who underwent surgical resection from January 2015 to June 2015 at the Department of thoracic surgery of the First Affiliated Hospital of Dalian Medical University. None of the patients had received preoperative adjuvant therapy after being diagnosed with primary lung adenocarcinoma. Informed consent was obtained from each patient, and the study was approved by the Ethics Committee of the Dalian Medical University.

### Cell culture

The human lung adenocarcinoma cell lines A549 and H1299 were purchased from the cell bank of the Committee on Type Culture Collection of the Chinese Academy of Sciences (Shanghai, China). A549 cells were cultured in F12K medium supplemented with 10% fetal bovine serum (FBS; Invitrogen, Tokyo, Japan). H1299 cells were maintained in RPMI-1640 supplemented with 10% FBS (Invitrogen). The human monocyte cell line THP-1 was also purchased from the cell bank of the Committee on Type Culture Collection of the Chinese Academy of Sciences. THP-1 were maintained in RPMI-1640 supplemented with 10% FBS (Invitrogen). All cells were cultured in cell-culture flasks or Petri dishes in a humidified incubator at 37˚C in an atmosphere of 5% CO2 [46].

### M2 macrophages conditioned medium

THP-1 cells were cultured with phorbol 12-myristate 13-acetate (20 ng/mL) and IL-4 (20 ng/mL) for 3 days for M2 macrophages polarization, as described in a published study. M2 macrophages derived form THP-1 cells were maintained in RPMI-1640 supplemented with 10% FBS (Invitrogen). The supernatants were collected and used as M2 macrophages conditioned medium (TCM). Different amount of TCM (0%, 25% and 50%) was added into complete medium to reach the final volume 1300 μL. The rest of volume was made up by FBS-free 1640.

### Cell transfection

FUT4-shRNA plasmids and FUT4-siRNA were designed and synthesized by Shanghai GenePharma Co., Ltd. (Shanghai, China). The sequences of FUT4 shRNA were as follows: 5′-GATCCGCCTGGCAAGT AACCTCTTCTCAAGAGAA AGAGGTTACTTGCCAG GCTTA-3′,5′-AGCTTAAAGCCTGGCAAGTAACCTCT TTCTCTTGAGAAGAGGTTACTTGCCAGGCG-3′. The WT Ezrin overexpression plasmids were designed and synthesised by Shanghai GenePharma Co., Ltd (Shanghai, China). The point mutants T567D Ezrin were obtained using the QuickChange mutagenesis kit (Stratagene, La Jolla, CA). All transfection reactions were performed using Lipofectamine 2000 (Invitrogen, Carlsbad, CA, USA) in accordance with the manufacturer's instructions. Stable transfectants were selected with 800 μg/ml G418 (Sigma-Aldrich, St. Louis, MO, USA), and individual clones were isolated.

### Immunohistochemistry

For immunohistochemical staining, the tissue specimens were incubated with antibodies to E-cadherin (ab 11512; 1:200 dilution; Abcam, Cambridge, UK), LeY (BG-8; 1:100 dilution; Abcam, Cambridge, UK) and CD68(ab955; 1:200 dilution; Abcam, Cambridge, UK). After washing, the sections were reacted with a biotin-conjugated secondary antibody and then incubated with a avidin-biotin-peroxidase complex. Finally, sections were visualized with DAB (3, 3Ndiaminobenzidine tetrahydrochloride). The immunoreactivities of immunohistochemical staining were assessed by scanning the whole tumor sections (Olympus IX81 microscope; Olympus, Tokyo, Japan). The immunoreactivities for E-cadherin and LeY was semiquantitatively scored by the cross-product (H-score) of the percentage of tumor cells staining at each of three staining intensities [47]. An H-score < 100 was considered “negative”, an H-score>100 was considered “positive”. We counted the mean number of macrophages (CD68) per field. A mean number < 20 was considered “negative” (−), a mean number > 20 was considered “positive” (+).

### TGF-β1 measurement with ELISA

Culture supernatants were harvested from M2 macrophages cells, centrifuged to remove cellular debris, and stored at −80°C. TGF-β1 protein levels were measured with a Human TGF-β1 ELISA Kit (ab100647, Abcam) according to the manufacturer's instructions. Each ELISA was carried out in duplicate for at least three separate experiments.

### Western blot analysis and immunoprecipitation

Proteins were separated by SDS-PAGE and transferred to nitrocellulose membrane (Bio-Rad, Hercules, CA, USA). Membranes were blocked in a buffer (TBS: 50 mM Tris-HCl, 150 mM NaCl, pH 7.4) containing 5% bovine serum albumin and 0.1% Tween-20, followed by incubation with the primary antibodys Phospho-Smad2 (Ser465/467)/Smad3 (Ser423/425)( D27F4, 1:1,000, CST), FUT4 (ab181461, 1:1,000, Abcam), LeY (BG-8,1:200, Abcam), N-Cadherin (ab18203, 1:1,000, Abcam), Fibronectin (ab2413, 1:2,000, Abcam), Vimentin (ab8978, 1:2,000, Abcam), ZEB1 (D80D3, 1:2,000, CST), Snail (ab53519, 1:2,000, Abcam), Ezrin (#3145, 1:2000, CST), UEA (LifeSpan BioSciences), p-Ezrin (Thr567) (#3726, 1:1000, CST) or β-actin (20536-1-AP, 1:2,000, Proteintech) diluted in the same buffer. The immunoreactive proteins were visualized using the ECL western blot analysis system (Bio-Rad), and densitometric analysis was performed using the Image Pro-Plus software.

For immunoprecipitation, the lysates were cleared using centrifugation at 16,000g for 10 min, incubated with protein A/G agarose beads and with anti Ezrin antibody (ab41672, 1:1,00, Abcam) at 4°C for 12–16 h. The beads were washed three times with lysis buffer and immunoprecipitates were separated from the beads by adding 2×ample buffer and boiled. SDS–PAGE-separated immunoprecipitates were transferred on PVDF membranes. The membranes were denatured with denaturation buffer containing 6 M guanidine chloride, 20 mM Tris (pH 7.5), 100 mM PMSF and 5 mMb-mercaptoethanol at 4°C for 30 min and washed three times with TBST. The membranes were blocked with 5% BSA and incubated with anti-LeY antibody (BG-8,1:200, Abcam).

### Immunofluorescence

Cells were seeded onto cover slips, fixed with 4% paraformaldehyde and permeabilized with 0.3% Triton x-100 for 10 min. Slides were blocked with 1% bovine serum albumin and incubated with E-cadherin (ab76055, abcam, 1:200), F-actin (ab205, abcam, 1:100) or β-catenin (ab32572, abcam, 1:200) antibodies overnight at 4˚C. After washing in PBS, the cells were stained with secondary antibodies and incubated for 1 h at room temperature, followed by nuclear counterstaining with DAPI.

### *In vivo* assays for tumor growth and metastasis

The *in vivo* tumorigenesis assay was performed, as followed. M2 or M0 macrophages were pre-mixed with A549 cells at a 1:4 ratio and were subcutaneously implanted into the left and right dorsal flank of 5-week-old female nude nice, respectively [20]. Growth curves were plotted, based on mean tumor volume at each time point, for each experimental group. The tumor dimensions were measured every 3 days using a digital caliper. The tumor volume (mm3) was calculated as follows: V = ab^2^/2, where a and b are the largest and smallest tumor diameters measured at necropsy, respectively. After 21 days, the mice were killed and the tumor tissues were harvested for use in further experiments.

### Statistics

All statistical analyses were done using the SPSS software package (version 13.0, SPSS Inc., Chicago, IL, USA). Data were shown as the mean ± s.d. of no less than three separate experiments. Relationships between E-cadherin, LeY, CD68 and clinicopathological variables were examined using χ^2^-test, respectively. Other data from experiments were analyzed using the Student's *t-test* or analysis of variance, as appropriate. A *P-value* < 0.05 was considered statistically significant in all cases.
